# Predictors of malaria-association with rubber plantations in Thailand

**DOI:** 10.1186/1471-2458-12-1115

**Published:** 2012-12-27

**Authors:** Pratana Satitvipawee, Warunnee Wongkhang, Sarika Pattanasin, Penprapai Hoithong, Adisak Bhumiratana

**Affiliations:** 1 Department of Biostatistics, Faculty of Public Health, Mahidol University, Bangkok, 10400, Thailand; 2 Center for EcoHealth Disease Modeling and Intervention Development Research, Faculty of Public Health, Mahidol University, Bangkok 10400, Thailand; 3 Chaiyarat Subdistrict Health Promoting Hospital, Bang Saphan Noi District, Prachuap Khiri Khan, 77170, Thailand; 4 Doctoral Program for Public Health (Epidemiology), Department of Epidemiology, Faculty of Public Health, Mahidol University, Bangkok, 10400, Thailand; 5 Prachuap Khiri Khan Provincial Health Office, Prachuap Khiri Khan, 77000, Thailand; 6 Department of Parasitology and Entomology, Faculty of Public Health, Mahidol University, Bangkok 10400, Thailand

**Keywords:** Health behavioral factors, Insecticide-treated nets, Long-lasting insecticidal nets, Malaria-associated rubber plantations, Occupational risk, Personal protection behaviors, Sleeping under mosquito-nets

## Abstract

**Background:**

The national Global Fund-supported malaria (GFM) program in Thailand, which focuses on the household-level implementation of vector control via insecticide-treated nets (ITNs)/long-lasting insecticidal nets (LLINs) combined with indoor residual spraying (IRS), has been combating malaria risk situations in different provinces with complex epidemiological settings. By using the perception of malaria villagers (MVs), defined as villagers who recognized malaria burden and had local understanding of mosquitoes, malaria, and ITNs/LLINs and practiced preventive measures, this study investigated the predictors for malaria that are associated with rubber plantations in an area of high household-level implementation coverage of IRS (2007–2010) and ITNs/LLINs (2008–2010) in Prachuap Khiri Khan Province.

**Methods:**

A structured questionnaire addressing socio-demographics, household characteristics and health behavioral factors (knowledge, perceptions and practices) regarding the performed interventions was administered to the 313 households (70 malaria-affected and 243 malaria-unaffected) that had respondents aged ≥18 years of both genders. In the univariate and multivariate analyses, only 246 (78.6%) MV respondents (62 malaria-affected and 184 malaria-unaffected) were analyzed to determine the predictors for risk (morbidity).

**Results:**

The majority (70%) of households were covered by IRS. For a combination of ITNs/LLINs, there were 74% of malaria-affected households covered and 46% of malaria-unaffected households. In a logistic regression analysis using odds ratios (aORs) adjusted on the variables and a 95% confidence interval (CI), malaria affecting MVs was associated with daily worker (i.e., earning daily income by normally practicing laborious activities mostly in agriculture such as rubber tapping and rubber sheet processing at the smallholdings of rubber plantations) (aOR = 2.9, 95% CI: 1.1-7.4), low-moderate level of malaria knowledge (aOR = 2.4, 95% CI: 1.1-5.0) and sleeping under mosquito-nets (nets/ITNs/LLINs intermittently and ITNs/LLINs only) (aOR = 2.0, 95% CI: 1.0-3.7).

**Conclusions:**

The MV predictors for malaria-association with rubber plantations included occupation (daily worker), misconceptions about malaria (mosquito and prevention) and the use of mosquito-nets. Human practices such as revisiting rubber plantations while exposed to multiple bites at multiple locations are more likely to apply to daily workers than to rubber farmers/tappers and others. The promotion and use of ITNs/LLINs depends substantially on cultural factors and defensive behaviors relevant to their occupational risk despite the perceived threats of malaria and the perceived benefits of ITNs/LLINs. This information supports the conclusion that GFM program implementation in Thailand or elsewhere for malaria-associated with rubber plantations would benefit from the potential use of ITNs/LLINs and changes in personal protection behaviors.

## Background

Thailand has a provisional service of food and agriculture products, which are influenced by the tropical monsoon climate in Southeast Asia [1]. The natural resources that people can use to earn income have become a matter of national importance due to the extreme land exploitation [2,3]. Several reports have shown that, in hilly areas that are either close to borders or are plantations located at tropical forest fringes with human settlements, agricultural practices of the population are often associated with malaria [4-8]. Notably, this forest-related malaria has continually caused mortality and morbidity, particularly in the Thai-Myanmar and Thai-Cambodia border provinces [9,10]. Malaria has becomes one of the most important mosquito-borne diseases in Thailand, and its transmission is caused by two main parasites, *Plasmodium falciparum* and *Plasmodium vivax*[11-14]. In endemic areas, seasonal malaria transmission exhibits a peak in the rainy season, during which most people who engage in agricultural practices are vulnerable [4,5,10].

With particular attention directed to the forested and border areas, the national malaria control program (NMCP) initially developed in 1971–1973 had emphasized two control measures: antimalarial drug treatment and anti-mosquito measures. The NMCP was revised with the adoption of the global malaria control strategies after 1995, which are both provisionally supported by the Global Fund to Fight AIDS, Tuberculosis and Malaria (GFATM) [15,16] and recommended by the World Health Organization (WHO) [17]. The new malaria control strategies have primarily focused on the use of immunochromatographic tests for rapid malaria diagnosis, artesunate combination therapy (ACT) for prompt treatment and indoor residual spraying (IRS) in combination with insecticide-treated nets (ITNs)/long-lasting insecticidal nets (LLINs) for vector control. As in other endemic countries implementing the Global Fund-supported malaria (GFM) program [18-21], the malaria burden in Thailand has dramatically declined domestically in terms of the overall malaria incidence rates. Groundbreaking bio-medical diagnostics and intervention technologies transcend the current knowledge of parasite-mosquito-human interactions, but community participation is the cornerstone of the household-level implementation of these pragmatic malaria control strategies across the country. Due to social segmentation and modern lifestyles, an effective mechanism employs malaria prevention/control campaign activities through public relations, health education and appropriately designed media, so that most people can comprehend every facet of the program or, at least, have increased awareness. Additionally, to enhance the effective management of the provincial GFM program, it is important to analyze information in different ways to capture different complex epidemiological settings resulting from socio-politic, socio-economic, demographic, technologic and environmental changes, or due to ineffective management of household-level information and improper implementation of those control strategies [17,19,22].

Prachuap Khiri Khan, a Thai-Myanmar border province, experiences malaria-sensitive situations because there is coexistence of cross-border population migrations (i.e., daily, periodic, seasonal and long-term) and internal movements of populations into hilly areas where housing and the use of land for plantations are unplanned. As one of the top-ten malaria-affected provinces [10], Prachuap Khiri Khan acts as a sub-recipient and has established malaria posts for the proactive implementation of the GFM such that free-of-charge ACT focuses predominantly on the people who are infected within the endemic villages or malaria transmission areas. This acts as a mainstay of malaria control strategy in supplement with the free distribution of highly subsidized ITNs/LLINs and IRS to at-risk households [17,22]. Because of a reduction of malaria-directed mortality and morbidity, the province expanded the distribution of ITNs/LLINs to the target population in the high transmission risk areas. Full coverage, including IRS combined with ITNs/LLINs for all people at risk, is ideal for malaria prevention, but in practical situations, such aid can only be provided to a number of vulnerable houses. Concurrently, an annually-fluctuating number of at-risk persons, including workers and farmers in agriculture, are either affected or re-threatened with malaria in different periods and at different places, despite the fact that the intervention services that they accessed were highly subsidized. Most likely, a person or family who work on rubber plantation are at the greatest risk of infection because they work outdoors at night and do not always sleep under mosquito nets [23]; however, the social factors and defensive behavioral factors prerequisite to change behavior and improve health practices [24-30] have never been established.

**Figure 1:**
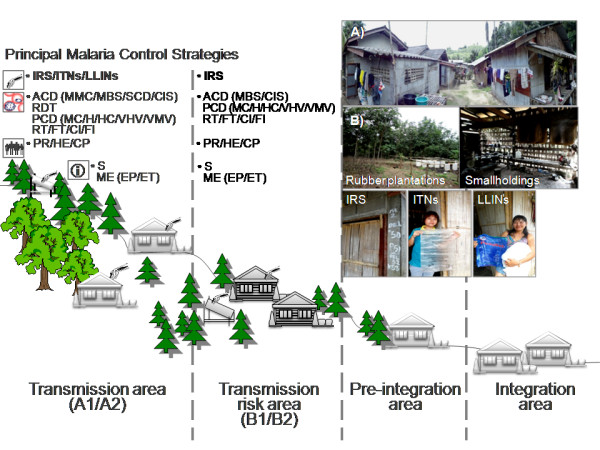
**Diagrams of malaria control stratification areas and strategies for a malaria-affected province of Thailand.** Malaria transmission area (perennial A1 and periodic A2) regularly occurs with indigenous malaria cases, while in transmission risk area (high-receptive B1 and low-receptive B2) introduced cases with a known infection orgin. With the absence of vectors and incidence for >3 consecutive years of control, the malaria-free zone targeted by the NMCP becomes pre-integrated and integrated into the basic health services in the province. Vector control strategies include IRS (regular and special spraying for A1/A2 as focal for B1/B2) and ITNs)/LLINs. Malaria chemotherapy focuses on both active (ACD) and passive (PCD) case detections, radical treatment (RT), follow-up treatment (FT), case investigation (CI) and foci investigation (FI). The ACD includes mobile malaria clinics (MMC), mass blood surveys (MBS), special case detection (SCD), case investigation surveys (CIS), rapid diagnostic testing (RDT) and ACT through malaria posts, as in the PCD, location and personnel aid the effort, such as the malaria clinic (MC), hospital (H), health center (HC), village health volunteer (VHV) and village malaria volunteer (VMV). For the behavior objective, strategic approaches employ public relations (PR), health education (HE) and community participation (CP). This NMCP management encompasses supervision (S) and monitoring and evaluation (ME) systems, both epidemiological (EP) and entomological (ET). At the household level, such malaria villagers (**A**) inhabiting transmission risk areas on rubber plantations (**B**) in Prachuap Khiri Khan Province that were covered by IRS (2007–2010) and ITNs/LLINs (2008–2010) were recruited into the study.

It is hypothesized that if there was a difference in local perceptions of mosquitoes, malaria, ITNs/LLINs and the practice of preventive measures among households, malaria infections and associated factors would vary between different epidemiological settings in Prachuap Khiri Khan. Thus, this analytical cross-sectional study conducted between January and April of 2011 investigated the predictors of the acquisition of malaria infections among malaria-endemic villagers inhabiting transmission risk areas on rubber plantations in the province (Figure 1). Understanding the undesired health behaviors that facilitate exposure to malaria is needed to design preventive measures, and, more specifically, to design individually adapted behavior interventions.

## Methods

### Study area and population backgrounds

The Moo 2 village, a study site that belongs to the Chaiyarat Subdistrict (6 villages in total), is situated in a malaria transmission risk area (Figure 1), 25 km southwest of the Bang Saphan Noi Hospital and District Health Office; both agencies are responsible for malaria control. Due to the subtle geographic disparities and topographic altitudes 100–200 meters above sea level, the village consists of 7 hamlets, namely Ban Chong Samkaew, Ban Hin Tern, Ban Kok Ai Poek, Ban Mak Poo, Ban Pong Toei, Ban Subsomboon and Nuay Anurak. The village covers uplands, hills, hillside slope areas and valleys. More than 80% of the villagers exploited their land for rubber and oil palm plantations and to a lesser extent for fruit orchards. A census of 435 households including 1,896 local inhabitants was officially reported after 2009 and was used in both pre-surveys of existing households in 2010 and random sampling as mentioned below. The majority of the population has access to electricity and running water system. Other water sources available for domestic use and agriculture are streams, brooks, shallow wells and reservoirs. The average number of rain days per year exceeds 120. The average annual temperature is 27.6°C with a maximum 41°C and a minimum 14°C.

### Existing malaria control measures and activities

**Figure 2:**
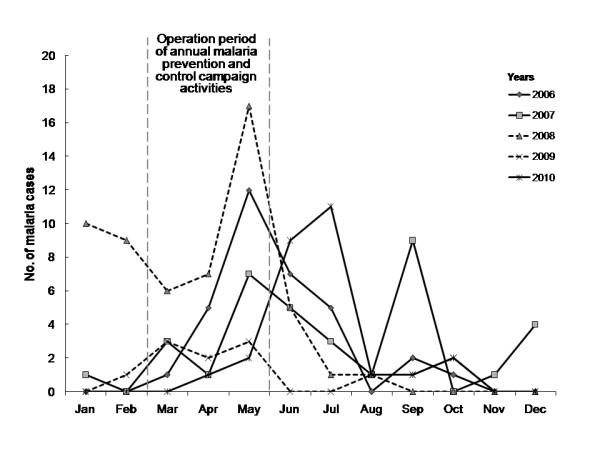
**The fluctuating trend of malaria incidence in the study village during the years 2006–2010.** A total of 130 malaria cases were reported. The established alliance corroborated malaria prevention/control campaign activities by which the affected malaria cases (dashed lines) are shown between years before (2008) and after (2009) horizontally implemented. Data were retrieved from the electronic reporting system for notifiable diseases maintained by Chaiyarat Subdistrict Health Promoting Hospital.

Supported by the GFM program since 2004 [9], Prachuap Khiri Khan Provincial Health Office has adopted the implementation of global malaria control strategies (RDT, ITNs/LLINs and IRS) at the provincial level (Figure 1) and has focused predominantly on reducing the mortality attributed to *P. falciparum* malaria in the 37 endemic villages (high-transmission A1/A2) (Figure 1). Subsequent to the initiation of operation coverage in the malaria transmission risk areas, the Moo 2 village effort commenced in mid-2008 because the number of malaria cases periodically reported for three consecutive years (2006–2008) had surged (Figure 2). The vector-borne disease control initiative centered not only on strengthening the capacity building of the communities, but also on promoting multisectoral partnerships under the supervision of the director of Bang Saphan Noi Hospital. The established alliance consisted of the Chaiyarat Subdistrict Administrative Organization, the Vector-borne Disease Control Unit (Bang Saphan) and two primary care units (formerly health centers), Chaiyarat Subdistrict Health Promoting Hospital and Ban Bang Charoen Subdistrict Health Promoting Hospital. The operational objective was to increase vector surveillance and control focused toward malaria prevention/control campaign activities that governed the household-level implementation of IRS and ITNs/LLINs together with public relations, community participation and health education, prior to and during the malaria transmission season.

According to WHO recommendations for active ingredient (ai) per square meter amounts [31], a 5% water-dispersible powder (WP) of deltamethrin at a concentration of 20 mg ai/m^2^ was used for IRS, and only one 25% water-dispersible tablet (WT) of deltamethrin at a concentration of 50 mg ai/m^2^ was used in treating mosquito nets or retreating ITNs. These measures can reduce the density of common indoor- and outdoor-biting vectors (*Anopheles minimus* and *An. maculatus*, respectively in South Thailand); however, nothing was known about the susceptibility of these vectors to effective doses of the insecticide used in the study area. Two trained village volunteers and malaria field workers distributed the ITNs/LLINs and performed focal IRS before (March-April) and during the peak (May-July) of the malaria transmission season (Figure 2).

### Sample size and selection of respondents

**Figure 3:**
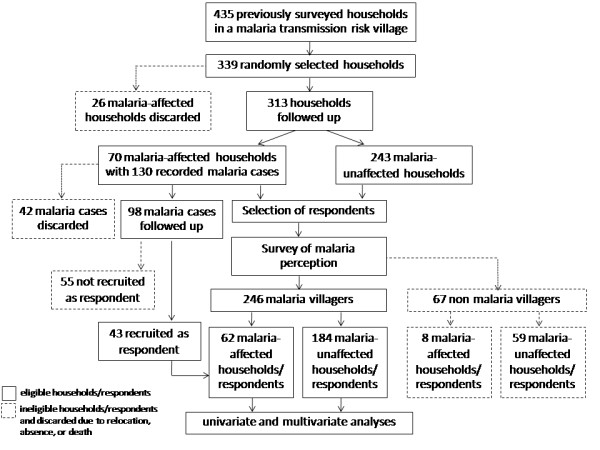
**Diagram displaying the successive processes of the selection of 313 households/respondents and 246 malaria villagers.** Malaria-affected households and malaria villagers are described in the text.

The data on malaria-affected households retrieved from the electronic reporting system of notifiable diseases revealed 130 malaria cases reported during the period between 2006 and 2010 (Figure 2). This information was used in sampling the households as follows. Of the 435 previously surveyed households (Figure 3), a statistically required sample size of 339 assigned to a stratified two-stage random sampling was calculated at a 95% confidence level. Hypothetically, this was based on the proportion of 20% malaria-affected households reported during this period and the estimate with a 10% of margin of error. However, the 26 malaria-affected households that had reported cases had been reported before 2007 were not available for selection due to either their relocation or absence. Finally, a total of 313 randomly selected households had knowledgeable respondents aged ≥18 years of both genders. These respondents (72% coverage) from the 7 hamlets were from 70 malaria-affected households and 243 malaria-unaffected households. A malaria-affected household was defined as any household that had at least one member who experienced malaria for the first time in his/her lifetime from 2007 to 2010, regardless of the type of infections (single or mixed), infection episode (once or multiple), relapsing of *P. vivax* malaria and malaria foci (inside or outside the village). In this regard, the at-risk households with any member involved in work at either rubber plantations or natural rubber productions who occasionally became infected with malaria might have been psycho-sociologically affected by the household member’s ailment. This was because the disease resulted in a deviation from a normal lifestyle and caused loss of work days. It directly reduced family income, indirectly increasing patient costs. Moreover, the family members felt anxiety about whether the malaria-infected member would spread the disease to others. Of the 70 malaria-affected households that were followed up with 75% (98/130) of recorded malaria cases (Table 1), only 44% of the follow-up cases (21 male and 22 female) were recruited into the study as the respondents; 25% were not followed due to relocation, absence or death (Figure 3).

**Table 1:** **A profile of the 98 malaria cases**^**a**^**from the 70 malaria-affected households**

**Clinical features**	**Male (n = 47)**	**Female (n = 51)**
Median years of age (IQR) and range	27 (12,45), 2-78	28 (14,50), 3-81
Single laboratory-confirmed infections^b^		
*P. falciparum*	29	27
*P. vivax*	15	21
No laboratory-confirmed infections^b^	3	3
Median days (IQR) and range of illness prior to hospitalization	3 (2,6), 1-7	3 (2,5), 1-9
Median days (IQR) and range of hospitalization	4 (2,5), 0-10	4 (2,7), 0-10

^a^All cases had their first infection between January 2007 and December 2010, and ^b^clinically were uncomplicated.

IQR, Interquartiles 25^th^ and 75^th^.

### Individual-level and household-level information

All 313 respondents were informed about the study objective and subsequently underwent face-to-face interviews using a structured questionnaire. Data on their socio-demographics and household characteristics, as well as on perceived burden of malaria and health behaviors regarding knowledge, perceptions and practices mentioned below, were recorded. The socio-demographic factors were gender, age, education, marital status, occupation, residence status and involvement in malaria prevention. Household characteristics included hamlet settlement, household economic status (monthly income and housing structure), surrounding environments, household-level implementation coverage of vector control measures (IRS and ITNs/LLINs) and utilization of mosquito-nets. Household economic status was categorized into 3 classes: monthly income ≤8,000 baht and poorly constructed house (low class), monthly income 8,001-15,000 baht and adequately constructed house (middle class), and monthly income >15,000 baht and well-constructed house (high class). IRS coverage at the household level from 2007 to 2010 depended on risk (morbidity). Some houses received IRS irregularly, only when malaria cases occurred within the hamlet, whereas in at-risk households, regular IRS (or focal spraying) was administered to reduce the density of *Anopheles* vectors prior to and during the malaria transmission season. Different households owned different types of mosquito-nets. Consequently, the use of mosquito-nets was categorized into 4 groups: non-use, sleeping under nets, sleeping under nets/ITNs/LLINs intermittently and sleeping under ITNs/LLINs only. The ITNs/LLINs implementation coverage for the at-risk target households began after 2008. The epidemiologic profile of this study population was similar to that of the Chaiyarat Subdistrict general population except that the household-level distribution coverage of IRS and ITNs/LLINs depended only on malaria risk. Moreover, data on general family health status, living conditions and environments were collected as a follow-up from and triangulated with databases of different sources, including family health folders and electronic health information systems accessed via Java Health Center Information System (JHICS) ver. 1.0. Ethical clearance and approval for the study (EC no. MUPH2010-180) was obtained from the Institutional Review Board at the Faculty of Public Health, Mahidol University. All respondents provided informed consent.

### Perceived burden of malaria and mapping

As noted earlier, the annual malaria prevention/control campaign activities (Figure 2), which are based on public relations, community participation and health education, had occurred in the village prior to the study. To evaluate the perception of malaria burden, all 313 respondents were, therefore, questioned about their knowledge of or hearing about malaria information through any information-conveying media and channels. In addition, they were questioned about whether they identified malaria as one of the top five public health problems affecting their family or the village community. Based on the survey responses, the 246 respondents who identified malaria as one of the top five public health problems affecting their family or the village community were labeled as malaria villagers (MVs), whereas the 67 remaining respondents who did not recognize malaria as a public health problem were labeled as non-MVs (Figure 3). These non-MVs were subsequently excluded from both the analysis of the proportions according to relevant health behavioral factors (knowledge, perceptions and practices) and the logistic model. For malaria mapping, the 70 malaria-affected households were asked to collect the coordinates (latitude and longitude) of their own houses and surrounding environments in the hamlets, using a global positioning system unit (eTrex Legend®, Garmin International, Inc., USA). This geographic information, by which their way-points were initially recorded in the field with a distance error ±10 meters, was transferred to a laptop running MapSource software version 6.15.7 and then manipulated using Google Earth maps. The spatial distributions of those premises within the studied village were displayed.

### Knowledge, perceptions and practices

The closed-ended structured questionnaire comprised three domains, which consisted of knowledge, perceptions and practices. The Cronbach's alpha coefficient of knowledge and perception was 0.7. The multiple-choice questions were used to ask about the cause, mode of transmission, vector and breeding place, diagnosis, clinical manifestations (symptoms, severity and cause of death), prevention and control of malaria to discriminate between misconceptions and accurate conceptions of malaria among the 246 MVs. Based on the judgments (i.e., agree, disagree and uncertain) of the MVs, the perceptions of malaria were related to health behavior factors, which included perceived susceptibility, severity, benefits and barriers of the health belief model [32].

Perceived susceptibility was assessed based on the responses to the following statements: 1) malaria is not serious; everyone gets infected; 2) a normal, healthy person is insusceptible to malaria; 3) a rubber farmer/tapper is at a greater risk of malaria than others; 4) more people are infected during the rainy season than during the summer season; and 5) drinking alcohol or smoking while doing outdoor activities at night can prevent bites from malaria vector. Perceived severity was assessed based on the responses to the following statements: 1) malaria is a severe disease, and the infected person can be re-infected; 2) malaria cannot be cured; 3) the first infection is more severe than re-infection; 4) malaria causes income and work day loss; and 5) malaria causes death unless treatment is administered. Perceived benefit was assessed based on the responses to the following statements: 1) neighbors of a malaria-affected house should receive ITNs/LLINs; 2) you should sleep under ITNs/LLINs only when the inhabitants of a house is affected by malaria; 3) both IRS and ITNs/LLINs should be implemented for a number of vulnerable houses in which a malaria case occurs; 4) regular IRS can reduce the density of mosquitoes or human-vector contact; 5) every family member should sleep under ITNs/LLINs to prevent malaria; and 6) sleeping under mosquito-nets can prevent malaria whenever staying at smallholdings in hilly areas on rubber plantations. Perceived barriers to control were assessed based on the responses to the following statements: 1) you are reluctant to allow village volunteers to operate IRS at your house; 2) treating or retreating mosquito-nets with insecticides should be the responsibility of health personnel/VHV/malaria workers; 3) sleeping under ITNs/LLINs is uncomfortable; 4) you do not need to own or use ITNs/LLINs if you or your family owns a smallholding in an area on a rubber plantation; 5) it is unsafe to sleep under ITNs/LLINs; and 6) you or a family member are willing to treat or retreat ITNs once or twice a year.

Practicing preventive measures at the household level is focused primarily on frequency of use (i.e., regular, irregular and never) of a combination of preventive measures (i.e., physical, chemical, electrical or fumigated) to repel or kill adult mosquitoes and thereby reduce human-mosquito contact, in addition to the use of screened windows that may act as a physical barrier in the house of the respondents. Finally, ratings for knowledge, perceptions and practices were based on the following scale: <60% low, 60-79% moderate and ≥80% good.

### Data analysis

The characteristics or individual explanatory factors for all 313 respondents (or 70 malaria-affected and 243 malaria-unaffected households) were described based on epidemiologic factors (i.e., socioeconomic, demographic and environmental) using descriptive statistics. In the initial univariate analysis, the chi-square test with Yates continuity correction (*P* < 0.05) on a 2x2 contingency table or Pearson’s chi-square test (*P* < 0.05) where appropriate, was used to analyze individual epidemiologic factor associated with malaria risk. Additionally, the household-level implementation coverage of the interventions, which included IRS (2007–2010) and ITNs/LLINs (2008–2010), was based on whether the at-risk households received either one of the intervention services once or twice a year over 4 consecutive years (2007–2010), regardless of the continuations of the interventions and the ITNs/LLINs owned per house or the number of persons who shared a net. Theoretical intervention effects might be related to complacency about malaria or to the people do not perceive the seriousness of the disease. Moreover, the utilization of mosquito nets was considered to be individually adapted behavior that the malaria cases may have practiced more routinely than the uninfected household members.

Similarly, all 246 MVs were used in the univariate analysis of the association between the health behavioral factors (knowledge, perceptions and practices) and malaria-affected MVs. The crude odds ratios (ORs) were computed to analyze the strength of their associations. In the multivariate analysis, the significant behavioral factors with ORs (*P* < 0.05 or *P* < 0.1) and those significant epidemiologic factors (*P* < 0.05) were included in a logistic model. The rates and adjusted odds ratios (aORs) for all of these variables and their 95% confidence intervals (CI) were calculated. The Wald’s test (*P* < 0.05) was used to test the statistical significance of each coefficient in the model to determine contributing predictors in a fitted model. Because a low number of cumulative malaria cases were reported in the study village, this multivariate analysis was performed collectively for the years 2007–2010 to estimate the effects of the behavioral factors on the implementation of malaria control strategies.

## Results

**Figure 4:**
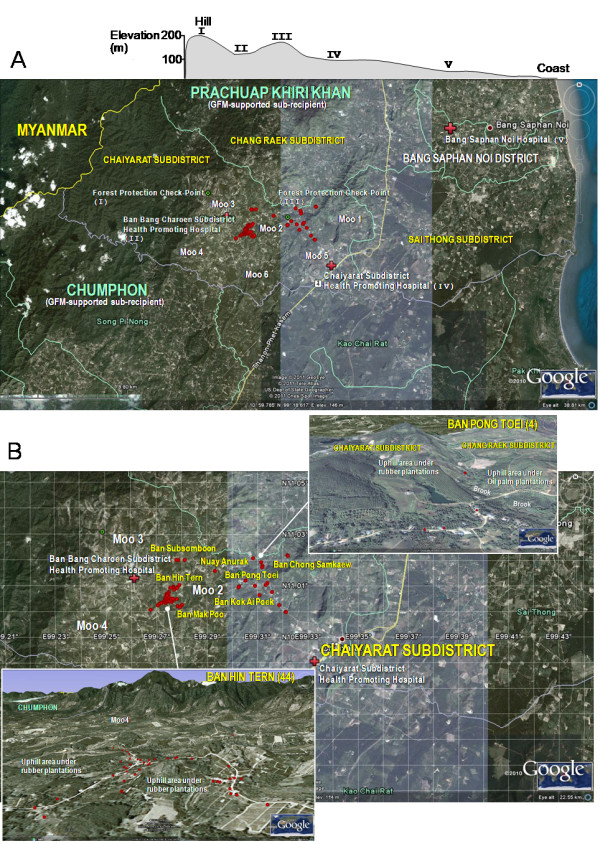
**Spatial distributions of all 70 premises with acquired malaria infections, 2007–2010.** (**A**) Endemic settings of the Chaiyarat Subdistrict and healthcare providers (red cross) in the Bang Saphan Noi District. Details include their elevation (m), from the hill (≥200 m) to the coast (10 m), 2 forest protection check-points (dotted green circle; I, 200 m, III, 185 m), 2 primary care units (II, 140 m; IV, 100 m), and a secondary healthcare facility (V, 50 m). (**B**) Distribution of all 70 malaria-affected households (red dot) in different hamlets of the study village Moo 2: two representatives (household numbers) are shown.

As illustrated in Tables 2 and 3, a total of 313 respondents consisting of 70 malaria-affected households and 243 malaria-unaffected households were used in the univariate analysis of the association between individual socio-demographic variable and malaria risk, as well as between household characteristic and malaria risk. Among the 70 malaria-affected households (Figure 4), the majority (62.9%) were living in Ban Hin Tern. Chi-square tests revealed that the following factors were significantly associated with malaria risk (details not shown): gender, occupation, residence status, hamlet settlement, household economic status, distance from the nearest road, distance from the nearest reservoir connecting brooks, ITNs/LLINs coverage and utilization of mosquito nets. Among these contributing factors, it was interesting to note that the intervention services (i.e., coverage of IRS and ITNs/LLINs) that were considered regarding the malaria risk tended to be proportional to the numbers of malaria-affected vs. unaffected households (Table 3). In total, 74.3% of malaria-affected households received the same ITNs/LLINs and IRS both irregularly and regularly. As expected, these expanded intervention services targeted nearby malaria-unaffected households, with approximately 66.7% covered by IRS and only 46.5% covered by ITNs/LLINs. More interestingly, the study villagers differed on their mosquito-net usage practices. All of the malaria-affected households utilized mosquito nets (100%), but different net types and usages were noted, with 47.1% sleeping under nets, 27.1% sleeping under nets/ITNs/LLINs intermittently and 25.8% sleeping under ITNs/LLINs only. In contrast, the malaria-unaffected households were likely to practice both non-use and use of different nets in that 63.0% used nets, 31.3% used both nets/ITNs/LLINs intermittently and ITNs/LLINs only, whereas 6% reported non-use.

**Table 2:** The univariate analysis of the association between socio-demographics and malaria-affected households (n = 313)

**Categorical variables**	**No. (%) of malaria-affected households (n = 70)**	**No. (%) of malaria-unaffected households (n = 243)**	***P*-value**
Gender			0.032^*^
Male	29 (41.4)	66 (27.2)	
Female	41 (58.6)	177 (72.8)	
Median years of age (25^th^, 75^th^ percentiles)	45 (33,53)	42 (32,53)	
Age group (years)			0.209
18-25	9 (12.8)	19 (7.8)	
26-60	55 (78.6)	188 (77.4)	
>60	6 (8.6)	36 (14.8)	
Marital status			0.892
Single	5 (7.1)	16 (6.6)	
Living with partner	51 (72.9)	172 (70.8)	
Divorced/widowed/separated	14 (20.0)	55 (22.6)	
Education level			0.068
Not educated^a^	17 (25.4)	37 (15.7)	
Primary (4–6 years of schooling)	43 (64.2)	152 (64.4)	
Upper than primary	7 (10.4)	47 (19.9)	
Occupation^b^			< 0.001^**^
Rubber farmer/tapper	35 (50.0)	150 (61.7)	
Daily worker	25 (35.7)	36 (14.8)	
Other occupations	10 (14.3)	57 (23.5)	
Residency status			0.002^*^
Native Thai villager	49 (70.0)	211 (86.8)	
Non-native Thai villager^c^	21 (30.0)	32 (13.2)	
Person having role in malaria prevention			0.761
Health personnel/village health volunteer	47 (67.2)	152 (62.5)	
Family head/member	11 (15.7)	49 (20.2)	
Local authority/village leader	7 (10.0)	20 (8.2)	
Do not know	5 (7.1)	22 (9.1)	
Perceived burden of malaria^d^			0.032^*^
Yes	62 (88.6)	184 (75.7)	
No	8 (11.4)	59 (24.3)	

^a^Of the 54, 20 native Thais and 34 non-native Thai villagers^c^ that were born either in Myanmar or Thailand. The majority were able to read and write.

^b^Two major occupational groups: rubber farmers/tappers (i.e., having private-owned smallholdings of rubber plantations in which they tapped the rubber trees and processed rubber sheets) and daily workers (i.e., earning daily income by performing labor activities mostly in agriculture such as rubber tapping and rubber sheet processing at the smallholdings of rubber plantations). The others included students, government employees and so on.

^d^Resulting survey responses: “Yes” referred to any person (labeled as MV) who identified malaria as one of top five public health problems affecting their family or the village community, as for “No” any person (labeled as non-MVs) who did not recognize malaria as a public health problem.

Statistically significant with ^*^Yates corrected χ^2^ test (*P* < 0.05), or ^**^Pearson’s χ^2^ test (*P* < 0.05), for two-independent samples.

**Table 3:** The univariate analysis of the association between household characteristics and malaria-affected households (n = 313)

**Categorical variables**	**No. (%) of malaria-affected households (n = 70)**	**No. (%) of malaria-unaffected households (n = 243)**	***P*-value**
Hamlet settlement			<0.001^*^
Ban Hin Tern	44 (62.9)	58 (23.9)	
Others	26 (37.1)	185 (76.1)	
Household economic status			0.016^**^
Low class	34 (48.6)	73 (30.0)	
Middle class	19 (27.1)	87 (35.8)	
High class	17 (24.3)	83 (34.2)	
Domestic animals present	(n = 65)	(n = 233)	0.154
Yes	33 (50.8)	93 (39.9)	
No	32 (49.2)	140 (60.1)	
Distance from the nearest road	(n = 64)	(n = 219)	0.015^*^
≤10 m	26 (40.6)	129 (58.9)	
>10 m	38 (59.4)	90 (41.1)	
Distance from the nearest reservoir connecting brooks	(n = 64)	(n = 219)	<0.001^**^
Absence within 500 m	29 (45.3)	41 (18.7)	
Presence ≤200 m	6 (9.4)	73 (33.3)	
Presence >200 m	29 (45.3)	105 (48.0)	
IRS coverage^a^			0.212
Not receiving	18 (25.7)	81 (33.3)	
Receiving irregularly	42 (60.0)	142 (58.4)	
Receiving regularly	10 (14.3)	20 (8.3)	
ITNs/LLINs coverage^b^			<0.001^*^
Not receiving	18 (25.7)	130 (53.5)	
Receiving	52 (74.3)	113 (46.5)	
Utilization of mosquito nets			0.004^**^
Non-use	0 (0.0)	14 (5.7)	
Sleeping under nets	33 (47.1)	153 (63.0)	
Sleeping under nets/ITNs/ LLINs intermittently	19 (27.1)	40 (16.5)	
Sleeping under ITNs/LLINs only	18 (25.8)	36 (14.8)	

Household-level coverage of IRS^a^ during years 2007–2010 and ITNs/LLINs^b^ during years 2008–2010 as described in the text. Statistically significant with ^*^Yates corrected χ^2^ test (*P* < 0.05), or ^**^Pearson’s χ^2^ test (*P* < 0.05), for two-independent samples.

As a result of the survey responses to the perception of malaria burden in the study village, a significant number of the 246 MVs that included 62 malaria-affected and 184 unaffected respondents were screened out of the 313 respondents (*P* = 0.032) (Table 2). The remaining 67 non-MVs: 8 malaria-affected and 59 unaffected respondents were excluded as outlined in this study. Only the MV respondents who recognized malaria burden, and likely, had observable health behaviors were necessary for the subsequent analysis. The number and percentage of MVs that responded correctly to questions regarding knowledge or responded properly to questions regarding perceptions and practices are presented in Table 4. Table 5 lists the overall scores for knowledge, perceptions and practices in regard to malaria prevention. Among the 246 MVs, the majority had low-moderate scores for perceived barriers (84.6%), practicing preventive measures (68.7%), knowledge of malaria (64.7%) and perceived susceptibility (61%), whereas good scores were achieved for perceived benefit (91.9%) and perceived severity (53.7%). The malaria-affected MVs had higher rates of low-moderate scores for knowledge (81%), perceived barriers (84%) and practicing preventive measures (81%). Because the effect of perceptions (susceptibility, severity, benefits and barriers) was important, we analyzed the odds ratio for each variable for malaria-affected versus malaria-unaffected MVs separately. These were calculated by using good scores as a reference (Table 5). The malaria-affected MVs who responded with low-moderate knowledge scores were at more than twice the risk (OR = 2.9, 95% CI: 1.1-4.9, *P* < 0.05) than those who had good knowledge. Similarly, those who responded with low-moderate scores of practicing preventive measures had significant risk (OR = 2.3, 95% CI: 1.4-6.2, *P* < 0.05), compared to those with good practices. In addition, we tested which aspects of malaria knowledge contributed to malaria risk. The results indicated malaria-affected MVs that had misconceptions about malaria had significant risks for malaria in regards to cause (OR = 2.2, 95% CI: 1.0-4.8, *P* < 0.05), vector (OR = 2.9, 95% CI: 1.5-5.4, *P* < 0.05) and prevention (OR = 2.2, 95% CI: 1.1-4.5, *P* < 0.05). The perceived susceptibility that interacted with malaria-affected MVs (*P* < 0.1) in regards to both gender and age, was kept only in the model.

**Table 4:** Knowledge, perceptions and practices of the 246 MVs

**Questions**	**No. of MVs (%) responding correctly or properly**		
	**Total (n = 246)**	**Malaria-affected (n = 62)**	**Malaria-unaffected (n = 184)**
**Malaria knowledge**			
Causation	70 (28.4)	11 (17.7)	59 (32.1)
Mode of transmission	234 (95.1)	60 (96.8)	174 (94.6)
Vector	157 (63.8)	28 (45.2)	129 (70.1)
Breeding place	98 (39.8)	19 (30.6)	79 (42.9)
Diagnosis	241 (98.0)	59 (95.2)	182 (98.9)
Clinical symptoms	240 (97.6)	61 (98.4)	179 (97.3)
Severity	169 (68.7)	46 (74.2)	123 (66.8)
Cause of death	133 (54.1)	28 (45.2)	105 (57.2)
Control	133 (54.1)	28 (45.2)	105 (57.2)
Prevention	196 (79.7)	43 (69.3)	153 (83.1)
**Malaria perceptions**^a^			
Susceptibility – Q1	120 (48.8)	24 (38.7)	96 (63.3)
Susceptibility – Q2	108 (43.9)	15 (24.2)	93 (50.5)
Susceptibility – Q3	168 (68.3)	43 (69.4)	125 (68.0)
Susceptibility – Q4	159 (64.6)	37 (59.7)	122 (66.3)
Susceptibility – Q5	128 (52.0)	31 (50.0)	97 (52.7)
Severity – Q1	147 (59.8)	37 (59.7)	110 (59.8)
Severity – Q2	115 (46.7)	24 (38.7)	91 (49.5)
Severity – Q3	65 (26.4)	12 (19.3)	53 (28.8)
Severity – Q4	230 (93.5)	57 (91.9)	173 (94.0)
Severity – Q5	204 (82.9)	51 (82.3)	153 (83.1)
Benefit – Q1	221 (89.8)	55 (88.7)	166 (90.2)
Benefit – Q2	197 (80.1)	46 (74.2)	151 (82.1)
Benefit – Q3	222 (90.2)	52 (83.9)	170 (92.4)
Benefit – Q4	208 (84.6)	47 (75.8)	161 (87.5)
Benefit – Q5	206 (83.7)	45 (75.8)	161 (87.5)
Benefit – Q6	111 (45.1)	26 (41.9)	85 (46.2)
Barrier – Q1	17 (6.9)	5 (8.1)	12 (6.5)
Barrier – Q2	175 (71.1)	43 (69.3)	132 (71.7)
Barrier – Q3	152 (61.8)	32 (51.6)	120 (65.2)
Barrier – Q4	180 (73.2)	45 (72.6)	135 (73.4)
Barrier – Q5	210 (85.4)	50 (80.6)	160 (87.0)
Barrier – Q6	138 (56.1)	35 (56.4)	103 (56.0)
**Practicing preventive measures**			
Physical	108 (43.9)	30 (48.4)	78 (42.4)
Chemical	65 (26.4)	11 (17.8)	54 (29.3)
Electrical	47 (19.1)	7 (11.3)	40 (21.7)
Fumigation	87 (35.4)	16 (25.8)	71 (38.6)

^a^The statements are described in the text.

**Table 5:** The univariate analysis of the association between health behavioral factors and malaria-affected MVs

**Health behavioral factors**^**a**^	**No. (%) of malaria-affected MVs (n = 62)**	**No. (%) of malaria-unaffected MVs (n = 184)**	OR^*^(95% CI)
Knowledge of malaria			
Low to moderate	50 (80.6)	109 (59.2)	2.89^*^ (1.38-6.17)
Good	12 (19.4)	75 (40.8)	
Perceived susceptibility			
Low to moderate	31 (50.0)	119 (64.7)	0.55^†^(0.29-1.02)
Good	31 (50.0)	65 (35.3)	
Perceived severity			
Low to moderate	25 (40.3)	89 (48.4)	0.72 (0.39-1.35)
Good	37 (59.7)	95 (51.6)	
Perceived benefits			
Low to moderate	8 (12.9)	12 (6.5)	2.12 (0.75-5.95)
Good	54 (87.1)	172 (93.5)	
Perceived barriers			
Low to moderate	52 (83.9)	156 (84.8)	0.93 (0.40-2.21)
Good	10 (16.1)	28 (15.2)	
Practicing preventive measures			
Low to moderate	50 (80.6)	119 (64.7)	2.28^*^ (1.08-4.87)
Good	12 (19.4)	65 (35.3)	

^a^Levels for knowledge and perceptions were based on rating scores <60%, low; 60-79%, moderate and ≥80%, good.

^*^OR, odds ratio; CI, confidence interval.

^*^P < 0.05 and ^†^*P* < 0.10 for the variables included in the model.

Table 6 displays the results of the logistic regression within each category of several socio-demographic and health behavioral factors that indicate associations between the predictor variables and malaria-affected MVs. The odds ratios for each variable were altered slightly when adjusted for the other variables, including gender and age. Among all the contributing factors tested, only the 3 predictors that included occupation, knowledge of malaria and utilization of mosquito-nets were associated with significant risk for malaria occurring among the MVs in the study village. The malaria-affected MVs were daily workers involved in rubber plantation work were more likely to experience a greater risk (aOR = 2.9, 95% CI: 1.1-7.4, *P* < 0.05), compared to those who were rubber farmers/tappers (aOR = 1.1, 95% CI: 0.5-2.5, *P* > 0.05) and those who performed other work. The malaria-affected MVs who had low-moderate knowledge scores were at a 2.4-fold significantly greater risk (aOR = 2.4, 95% CI: 1.1-5.0, *P* < 0.05) than those who had good knowledge scores. With regard to the utilization of mosquito-nets, the malaria-affected MVs who slept under nets/ITNs/LLINs intermittently and ITNs/LLINs only were twice as likely to present a greater risk (aOR = 2.0, 95% CI: 1.0-3.7, *P* < 0.05) than those who slept under nets and did not sleep under nets.

**Table 6:** Risk determinants that influenced malaria infections among the MVs

**Determinants**	**Total MVs (n = 246)**	**Malaria-affected MVs (n = 62)**	**%**	**Crude OR**^‡^**(95% CI)**	**OR**^†^**(95% CI)**
Occupation					
Daily worker	51	23	45.1	3.96^*^ (1.58-10.07)	2.92^*^ (1.14-7.44)
Rubber farmer/tapper	146	29	19.9	1.33 (0.59-3.08)	1.06 (0.45-2.48)
Others	49	10	20.4	1.0	1.0
Knowledge of malaria					
Low to moderate	159	50	31.4	2.89^*^ (1.38-6.17)	2.35^*^ (1.11-4.99)
Good	87	12	13.8	1.0	1.0
Practicing preventive measures					
Low to moderate	169	50	29.6	2.28^*^ (1.08-4.87)	1.71 (0.80-3.64)
Good	77	12	15.6	1.0	1.0
Utilization of mosquito-nets					
Sleeping under nets/ITNs/LLINs intermittently and ITNs/LLINs only	95	36	37.9	2.93^*^ (1.56-5.54)	1.96^*^ (1.03-3.72)
Sleeping under nets and non-use	151	26	17.2	1.0	1.0

^‡^OR, odds ratio; CI, confidence interval.

^†^Adjusted by the variables, gender and age, which is included in the model. ^*^P < 0.05.

## Discussion

An ample supply and distribution of mosquito nets, commonly ITNs, to at-risk populations and the promotion of sleeping under mosquito nets and their proper use, are the central components of malaria prevention and control [17]. Based on empirical evidence in Africa, the proper use of ITNs results in a reduction of malaria-directed mortality and morbidity, particularly in the children under 5 years of age and pregnant women [23,33-41]. It is clear that the benefits of using mosquito nets to significantly reduce malaria-directed deaths are enhanced not only by treating/retreating mosquito nets with insecticides, but also by increasing access to ITNs [42,43]. It has been observed that the pyrethroid insecticides that are used for both ITNs/LLINs and IRS have an excito-repellent effect. Commonly, the efficacy of these operational vector control interventions, if implemented properly and with coverage of almost the entire target population, protect the entire at-risk population [39,44-46] because the interventions reduce the density of indoor-biting *Anopheles* mosquitoes [47,48] and hence diminish human–vector contact and their vectorial capacity to transmit malaria parasites. IRS implemented by the endemic countries depends on the existing available resources and the management of vector control services. The effective and sustained implementation of IRS in combination with ITNs/LLINs depends greatly on complex stakeholder involvement between the affected communities and different levels of health agencies, networks and local authorities.

Therefore, a contemporary agendum for the country-specific issues guided by the WHO [17-22] has focused on scaling-up the implementation of IRS and ITNs/LLINs and building collective management solutions. For instance, Thailand is concentrating not just on how to monitor and evaluate the effective performance of the GFM program at the provincial level and whether it meets the outcome indicators (i.e., a reduction in the level of malaria-related mortality/morbidity) but also on how to design the individually adapted behavior interventions complementary to extending the coverage of ITNs/LLINs that only the at-risk populations fully access. For the latter goal, the difficulties include how to understand the processes that familiarize general versus at-risk populations with certain health practices and preventative actions. Ideally, risk reduction depends not only on the at-risk household that has full accesses to IRS and ITNs/LLINs but also on the proper uses of mosquito nets by every family member; no one should have occupational risk. We hypothesized that, in the study village of malaria-associated rubber plantations, the infected MVs who had misconceptions and negative perceptions might neither have individually adapted to sleeping-under-nets nor routinely practiced preventive measures against outdoors bites at night from *Anopheles* mosquitoes, regardless of zoophylaxis. As a result of the multivariate analysis, only the 3 significant determinants as major contributing predictors to the acquisition of malaria are debated below, with regards to the performance of the GFM program recently deployed into the study village. The perceptions and practices regarding malaria prevention did not demonstrate a significant effect in both the univariate and multivariate analyses. To capture the requisite data on health behavioral factors as the foundations of a process of behavioral change, the factors are also discussed.

### Coverage of IRS and ITNs/LLINs

Regular IRS (or focal spraying) is aimed at reducing the density of *Anopheles* mosquitoes within at-risk households. This service also interrupts transmission within a number of houses when any malaria case is reported. Most study households covered by IRS services in the 4 past years were due to the unstable case morbidity in the study village. Similarly, a number of ITNs/LLINs were allocated freely to at-risk households to help vulnerable persons. In the study village, there must have been expansion of the combined intervention services to the target households, both the malaria-affected households and nearby malaria-unaffected households. As expected, all malaria-affected households that had access to IRS received ITNs/LLINs. Markedly, two-third of malaria-unaffected households covered by IRS received ITNs/LLINs. Some malaria-affected households, or even nearby malaria-unaffected households, particularly those uncovered by IRS and ITNs/LLINs are of interest. When the perceived barriers to implementation were examined, it was noted that the MVs felt reluctant to allow village volunteers or malaria field workers to operate IRS at their house; this may account for many households uncovered by IRS and ITNs/LLINs, as seen in Table 3. Moreover, both groups reduced the use of ITNs/LLINs because not all households that owned ITNs/LLINs used them, although almost the entire MV group believed in the potential benefits of ITNs/LLINs. The cultural factors that determine intra-allocation, ownership, retention and the use of ITNs/LLINs are considered to be significant [23,49-52]. We found that, as shown in Table 3, most malaria-affected households that owned ITNs/LLINs might have individually adapted the use of ITNs/LLINs because they used both nets/ITNs/LLINs intermittently and ITNs/LLINs only, whereas there were no reports of non-use. Similarly, most malaria-unaffected households that owned ITNs/LLINs neither used ITNs/LLINs nor slept under mosquito-nets, suggesting that cultural factors also determined sleeping-under-net behaviors. Therefore, in agreement with the observations of the perceived benefits of ITNs/LLINs, sleeping under mosquito nets, particularly ITNs/LLINs, was considered a positive-protecting behavior. However, there were some counterintuitive problems that most study households both unaffected and affected with malaria that owned ITNs/LLINs did not use them all year round whether their houses were treated with IRS before or during the peak of seasonal transmission.

Generally speaking, our findings were in agreement with previous findings [32,49-52] in that we found two main social factors for the non-use of ITNs/LLINs. The reasons were that the rectangular ITNs/LLINs owned were not large enough, i.e., neither appropriate for mother/husband who shared with children nor uncomfortable for adult persons who slept and that they were kept for the relatives or visitors who stayed at their houses. When questioned about the perceived barriers of the implementation, most MVs mentioned the individual or household role in treating/retreating the mosquito nets. Unlike complacency, the MVs felt that ITNs/LLINs were uncomfortable and unsafe for sleep. The MVs felt that they needed neither to own nor use ITNs/LLINs if they owned a smallholding in the area on a rubber plantation. This perception could explain why the MVs that received ITNs/LLINs did not use them or had intra-allocation of ITNs/LLINs with not everyone sleeping under ITNs/LLINs despite the perceived threat of malaria.

As expected, the household members who slept under the mosquito nets, particularly ITNs/LLINs, were more likely to be vulnerable in that they perceived of the risk only when any member developed malaria illness, and the persons that had experienced malaria in the past or recently practiced good behaviors more routinely than those who had not been infected. Thus, for example, the ITNs/LLINs owned by these study households were more likely to be used as directed by the village health volunteers and local health personnel than as practiced by their motivation or readiness [32] because of their concern about the benefits of ITNs/LLINs. Similar to the observations of the perceived benefits of IRS and ITNs/LLINs, the individually adapted behavior was considered a significant trade-off because the mosquito nets, including ITNs/LLINs, generally used in the study village were felt to be effective against malaria [49-52]. This may be a reason why, in the model, the utilization of mosquito-nets (i.e., sleeping under nets/ITNs/LLINs intermittently and ITNs/LLINs only) had a significant association with malaria among the malaria-affected MVs. Nonetheless, it was not guaranteed that the greater increase in ITNs/LLINs coverage was related to the smaller decrease in a number of malaria cases in the transmission risk area on rubber plantations.

### Socio-demographic

In the study village with malaria-associated rubber plantations, it was clear when the household members likely came into close contact with various bites of *Anopheles* mosquitoes based on their nighttime activities. Some vulnerability in how either a person or family acquired the infections depended on household members being involved with rubber tapping in rubber plantations at night and also with rubber-sheet processing in smallholdings both during the night and day, although a high coverage of IRS and ITNs/LLINs at the household level had been achieved. Generally when examined for the perceived susceptibility of malaria, the MVs regarded malaria acquisition as rubber farmer/tapper as an occupational risk. However, in the model, the MVs who were daily workers were at a higher risk than rubber farmers/tappers for malaria infection. Most daily workers settled in Ban Hin Tern, and generally, came from households of low to middle economic class. Notably, daily workers were hired to earn wages by job opportunity, and occasionally, they came into close contact with various *Anopheles* mosquitoes during different periods and at different places during rubber tapping and rubber-sheet processing in the hilly areas of rubber plantations, particularly when working in high endemic Moo 3 and Moo 4 villages (data not shown) (Figure 3). It is unlikely that most rubber farmers/tappers who worked at night in smaller rubber plantations at one place might have a lower chance of being subjected to infective bites of *Anopheles* mosquitoes. Perhaps the activities of these vulnerable persons such as revisiting more rubber plantations could have exposed them to multiple bites at multiple locations in the study village. Further investigation of this possibility is needed. Recently, the malaria incidence in the study village responsible for high transmission (A2) has resulted in the 2011 establishment of a malaria post in Ban Hin Tern.

### Malaria knowledge

The MVs, as outlined in this study, recognized malaria burden in the affected study village, implying that they should have increased malaria knowledge and awareness through the prevention/control campaign activities. With regards to the aspects of malaria knowledge, the MVs that had good knowledge scores were able to comprehend the mode of transmission (night biting of malaria vectors), diagnosis (blood examination) and clinical manifestations (symptoms). The MVs realized that the blood examination should be performed in a timely manner if an individual developed flu-like symptoms (fever, headache and myalgia) during the rainy season. These figures may imply a perceived health threat [30,53]. In addition, the median days of illness prior to hospitalization and hospital adherence were 3 and 4 days respectively for both genders as seen in Table 1. In contrast to self-medication [53], the MVs sought medication as soon as they recognized malaria-like onset fever, suggesting that their household members with prodromal infections were less likely to delay treatment onset and that the case management was likely to be effective. However, our observations demonstrated that most MVs were more likely to have low-moderate knowledge scores than good knowledge scores. Similar to previous studies that investigated other malaria-endemic villagers [54], care takers of children under 5 years of age [26,53] and pregnant women [30], the malaria-affected MVs had misconceptions about malaria regarding the cause, vector and prevention. These figures were in agreement with the observations of malaria perceptions. The malaria-affected MVs claimed that, in hilly areas on rubber plantations, mosquito density in the rainy season was higher than in the summer season when night-biting mosquitoes were thought to be a nuisance but were not considered dangerous [55] despite the perceived threat of malaria. This might substantially contribute to malaria risk in the model. A low-moderate level of malaria knowledge was significantly associated with the malaria-affected MVs whose misconceptions regarding mosquitoes and prevention might influence the promotion and use of mosquito nets and other preventive measures. However, it is unwise to suggest that these results indicate the pitfalls of a health education program that makes use of information, education and communication strategies or, rather, a perceived failure of malaria prevention/control campaign activities that preceded the study in the village.

### Practicing preventive measures

Our study did not indicate a positive association between practices regarding malaria prevention and malaria, but, as seen in Table 4, our study indicate the MVs who had low-moderate practice scores might have practiced behaviors that increased exposure frequency of outdoor bites of *Anopheles* vectors in the study village. Clearly, the malaria-affected MVs had practiced preventive measures less frequently than the malaria-unaffected MVs, thus, the malaria-affected MVs who had low-moderate practices likely played a more significant role than those who had good practices. Additionally, the underlying factors for certain health practices that the MVs individually adapted were based on the severity of malaria illness symptoms they recognized, media campaigns they comprehended but not prolonged learning, advice from relevant people (family, village health volunteers and health staff) and the availability of preventive measures that they could afford. This figure may reflect the availability of individually adapted interventions applied in the study village. The MVs were more likely to practice both regularly and irregularly the combined preventive measures rather than just one; in this regard, the selection of preventive measures differed from one household to another. For examples, most MVs used an electric fan/mosquito coil or spray/mosquito repellent lotion or talcum powder, or fumigated natural plant products, as well as used electronic mosquito trap/electric mosquito repellents with a repellent mat or repellent liquid refill, before bedtime or during outdoor activities. In addition, they lit their own houses inside or outside all night. These findings are consistent with malaria knowledge in that the MVs commonly thought that the combined preventive measures were more effective against malaria than only sleeping under nets/ITNs/LLINs. Neither the protective effects of preventive health practices nor the internal and external factors and how they could influence different actions have been elucidated.

## Conclusions

The GFM program expanded distribution of the highly subsidized IRS and ITNs/LLINs to the households in malaria transmission risk areas on rubber plantations of the study village. As a result of the expansion of these intervention services, the MVs had local perceptions of mosquitoes, malaria, ITNs/LLINs and were practicing preventive measures, based presumably on the conveyed information that they gained from different sources. The predictors that determine the link between malaria and the affected MVs in such an ecotope are the occupation (daily worker), misconceptions about malaria (i.e., vector information and prevention) and the use of mosquito nets (i.e., sleeping under nets/ITNs/LLINs intermittently and ITNs/LLINs only). Rather, the malaria-affected MVs perceived the benefits of the use of mosquito nets due to increases in morbidity and the coverage of ITNs/LLINs. Clearly, the majority of MVs demonstrated a strongly perceived threat of malaria and perceived the benefits of ITNs/LLINs, but the promotion and use of ITNs/LLINs depended greatly on cultural factors and negative-protecting behaviors relevant to their occupational risk. This information supports the conclusion that GFM program implementation in different provinces with local variability in Thailand or elsewhere for malaria-associated rubber plantations would benefit from the potential use of ITNs/LLINs, and if implemented simultaneously, changes in personal protection behaviors.

## Competing interests

The authors declare that they have no competing interests.

## Authors’ contributions

PV conceived the study design, conducted the investigation, analyzed the data, drafted the manuscript and contributed to the revised manuscript. WW and SP participated in the conception and collected, managed and analyzed the data. PH supervised the study and contributed to the revised manuscript. AB conceived of the study design, conducted the investigation, analyzed the data, and drafted, revised and edited the manuscript. All authors read and approved the final manuscript.

## Pre-publication history

The pre-publication history for this paper can be accessed here:

http://www.biomedcentral.com/1471-2458/12/1115/prepub
